# Roles of 5-Lipoxygenase and Cysteinyl-Leukotriene Type 1 Receptors in the Hematological Response to Allergen Challenge and Its Prevention by Diethylcarbamazine in a Murine Model of Asthma

**DOI:** 10.1155/2014/403970

**Published:** 2014-11-11

**Authors:** Daniela Masid-de-Brito, Túlio Queto, Maria Ignez C. Gaspar-Elsas, Pedro Xavier-Elsas

**Affiliations:** ^1^Departamento de Imunologia, Instituto de Microbiologia Paulo de Góes, Universidade Federal do Rio de Janeiro, 21.941-590 Rio de Janeiro, RJ, Brazil; ^2^Departamento de Pediatria, Instituto Nacional de Saúde da Mulher, da Criancça e do Adolescente Fernandes Figueira, FIOCRUZ, 22.250-020 Rio de Janeiro, RJ, Brazil

## Abstract

Diethylcarbamazine (DEC), which blocks leukotriene production, abolishes the challenge-induced increase in eosinopoiesis in bone-marrow from ovalbumin- (OVA-) sensitized mice, suggesting that 5-lipoxygenase (5-LO) products contribute to the hematological responses in experimental asthma models. We explored the relationship between 5-LO, central and peripheral eosinophilia, and effectiveness of DEC, using PAS or BALB/c mice and 5-LO-deficient mutants. We quantified eosinophil numbers in freshly harvested or cultured bone-marrow, peritoneal lavage fluid, and spleen, with or without administration of leukotriene generation inhibitors (DEC and MK886) and cisteinyl-leukotriene type I receptor antagonist (montelukast). The increase in eosinophil numbers in bone-marrow, observed in sensitized/challenged wild-type mice, was abolished by MK886 and DEC pretreatment. In ALOX mutants, by contrast, there was no increase in bone-marrow eosinophil counts, nor in eosinophil production in culture, in response to sensitization/challenge. In sensitized/challenged ALOX mice, challenge-induced migration of eosinophils to the peritoneal cavity was significantly reduced relative to the wild-type PAS controls. DEC was ineffective in ALOX mice, as expected from a mechanism of action dependent on 5-LO. In BALB/c mice, challenge significantly increased spleen eosinophil numbers and DEC treatment prevented this increase. Overall, 5-LO appears as indispensable to the systemic hematological response to allergen challenge, as well as to the effectiveness of DEC.

## 1. Introduction

There is considerable evidence that eosinophils, a prominent feature in the characteristic inflammatory infiltrates of immediate hypersensitivity reactions, and of related chronic conditions, including allergic asthma [1–3], play a pathogenetic role by releasing granular cytotoxic proteins, cytokines, and lipid mediators [4–6]. In acute eosinophilic inflammation, infiltrating eosinophils eventually die through apoptosis and are cleared by resident macrophages, leading to resolution [7]. By contrast, a sustained increase in bone-marrow eosinophil production (*eosinopoiesis*), as well as prolonged survival in the peripheral inflammatory site, is believed to promote chronic allergic inflammation in humans and mice [8–10]. Furthermore, there is evidence that eosinophil progenitors accumulate in the challenged lungs of sensitized mice, suggesting that extramedullary eosinopoiesis also contributes to the hematological response to allergen exposure [11, 12].

There is interest in characterizing the mechanisms that ensure the selective increases in eosinopoiesis, both inside and outside the bone-marrow, following exposure to allergen challenge in sensitized subjects. These mechanisms were initially shown to act systemically, because the bone-marrow is not directly exposed to allergen but responds to factors generated in the lungs, which are demonstrable in plasma by a transfer protocol [9]. More recently, TNF-*α* and corticosterone, a stress hormone released by the adrenal glands, were shown to be required for the increase in eosinopoiesis in response to allergen challenge of sensitized mice [10]. However, neither TNF-*α* nor corticosterone is eosinophil-selective in their effects, and their production is not restricted to sensitized/challenged animals. This highlights the need to identify additional coupling elements which could account for the eosinophil-selective response in bone-marrow or in sites of extramedullary hemopoiesis. Here we have examined whether the 5-lipoxygenase (5-LO) pathway plays a role in the hematological response to allergen challenge, a possibility which is suggested by numerous observations, clinical and experimental.

5-LO generates a wide variety of mediators, through the action of specialized terminal enzymes variously expressed in different cell types, which act on the initial 5-LO products and their immediate derivatives, like leukotriene (LT) A4, to yield leukotriene B4 and the cysteinyl-leukotrienes (CysLT), LTC4, LTD4, and LTE4 [13–15]. There is evidence of an important role of CysLT in the pathophysiology of asthma and other allergic diseases, consistent with the clinical benefits of blocking their synthesis or their actions [13–15]. Cells expressing 5-LO are present in bone-marrow, and hemopoietic cells from both bone-marrow and other sites respond to 5-LO products, especially to CysLT [16–18]. Eosinophils both produce and respond to CysLT [4]. In bone-marrow cultures stimulated by interleukin (IL)-5, the major eosinopoiesis-promoting cytokine and lineage-specific survival factor [1–3, 7, 17], exogenously added CysLT, significantly enhance eosinopoiesis [19, 20]. Furthermore, type 1 CysLT receptors (CysLT1R) mediate the enhancing actions of the nonsteroidal anti-inflammatory drugs, indomethacin and aspirin [19], and of the proallergic cytokines, eotaxin/CCL11 and interleukin (IL)-13 [20], on eosinopoiesis. Finally, CysLT protects developing eosinophils from the proapoptotic effects of various mediators of inflammation, including prostaglandin (PG) E2 [16] and interferon- (IFN-) *γ* (Gaspar-Elsas, Queto et al., submitted).

Even though IL-5 signals through a common *β* chain (*β*c), which is also used by GM-CSF and IL-3 to signal through their own receptors, IL-5, unlike the other cytokines in this group, is preferentially expressed in the eosinophil lineage and is necessary for physiological eosinopoiesis [21]. Hence, interactions between IL-5 and 5-LO products* in vivo* could promote a lineage-specific hematological response to allergen challenge. Although the observations in bone-marrow culture suggest this possibility, they were made with bone-marrow from naive mice, after addition of exogenous agents (CysLT; NSAID; cytokines). On the other hand, suggestive evidence was obtained in a murine model of asthma, through the demonstration of a beneficial effect of diethylcarbamazine (DEC), an antifilarial drug [22]. DEC, known to suppress leukotriene synthesis [23], abolishes the eosinopoietic response to allergen challenge in sensitized mice, as well as eosinophil infiltration in the challenged lungs [24, 25]. This observation pointed to the possibility that leukotrienes, produced* in vivo* after challenge, contribute to the hematological response in these conditions and that inhibition of leukotriene synthesis by DEC underlies its effectiveness. If so, similar effects should be demonstrable in animals submitted to blockade or inactivation of the 5-LO pathway, independently of DEC.

This hypothesis was tested in sensitized and challenged wild-type mice of different strains, as well as in mutants lacking 5-LO, by evaluating the effectiveness of various drugs capable of interfering with leukotriene synthesis, or with CysLT1R signaling, to prevent the bone-marrow response to allergen exposure. In addition, we examined the effects of sensitization and challenge on the accumulation of eosinophils in the spleen, as well as the effectiveness of DEC in preventing this component of the hematological response to challenge.

## 2. Methods

### 2.1. Reagents

FCS was from Hyclone (Logan, UT); culture media RPMI 1640 from RHyClone, Thermoscientific, (Waltham, MA); recombinant murine interleukin-5 (IL-5) from R&D Systems (Minneapolis, MN, USA); grade II ovalbumin (Cat. A5253), methylcellulose (Cat. M0387) from Sigma (St. Louis, MO, USA); aluminium ammonium sulfate [AlNH_4_(SO_4_)_2_ *·*12H_2_O, alum] (Cat. 01S1048.01.AF) (Manufacturer, Synth, Brazil), grade V ovalbumin (Cat. 950 512), from ICN Biomedicals (USA); and MK886 (Cat. 10133), montelukast (Cat. 10008318), from Cayman Chemical (USA).

### 2.2. Animal Suppliers and Ethical Aspects

Wild-type mice of the BALB/c and 129S2/SvPas (PAS) strains and 129S2/SvPas-Alox (ALOX) mutants lacking functional 5-lipoxygenase genes [26], bred at CECAL-FIOCRUZ, Rio de Janeiro, Brazil, were used at 6–8 weeks of age for sensitization, challenge, and drug treatment experiments* in vivo* and as a source of bone-marrow cells for* ex vivo* analyses, following institutionally approved (CEUA-FIOCRUZ # L-010/04 and CEUA-FIOCRUZ# L-002/09, and CEUA-CCS-UFRJ 181) protocols.

### 2.3. Animal Procedures

Mice were sensitized with two subcutaneous injections of ovalbumin (100 *μ*g ovalbumin mixed in 1.6 mg alum in a total volume of 0.4 mL saline), 7 or 14 days apart [24]. The animals received one* intranasal *challenge (10 *μ*g grade V OVA/25 *μ*L of saline) on day 14 or three* intranasal* challenges (25 *μ*g grade V OVA/25 *μ*L of saline) on days 19, 20, and 21. Alternative challenge protocols were used in selected experiments:* aerosol *challenge at day 14, once, for 1 h, with 2.5% grade II ovalbumin in PBS, and* intraperitoneal* challenge, once, at day 14, with 10 *μ*g grade V OVA/400 *μ*L of saline. In selected experiments, mice were given MK886 orally, 1 mg/kg, in 0.1% methylcellulose/deionized water, on days 13 and 14, the latter dose being administered 1 h before challenge. Controls received methylcellulose vehicle. In selected experiments, mice were given DEC orally, 12 mg/kg, in deionized water, for 12 days beginning at day 14 or at days 19, 20, and 21, with DEC treatment 2 h before each daily challenge. Controls received the same volume of vehicle [24]. In selected experiments, mice were given montelukast orally, 10 mg/kg, in 2% DMSO/PBS 1X, administered 1 h before challenge. Controls received 2% DMSO/PBS 1x vehicle.

### 2.4. Sample Collection

Animals were submitted to euthanasia in a CO_2_ chamber.* Peritoneal lavage fluid* was collected after washing 3x the peritoneal cavity with 10 mL of chilled RPMI1640 medium (serum-free) using a 22G needle [26]. Recovery was typically 8-9 mL of the injected volume. The sample was centrifuged and the cell pellet were resuspended in 2 mL of the same medium with 1% FCS, for total (after dilution in Turk's stain) and differential (after staining for eosinophil peroxidase; [27, 28]) counts on haemocytometer and cytocentrifuge slides, respectively. Spleen cells were prepared as single cell suspensions from individual spleens where indicated, by mincing the spleen with scissors and needles and repeatedly passing the cell suspension through a syringe with no needle attached. Numbers of total nucleated cells and eosinophils were determined by hemocytometer and cytocentrifugate counts, as above.

### 2.5. Bone-Marrow Studies

Bone-marrow cells were collected from both femurs of individual naïve mice, washed, counted in a haemocytometer, seeded at 10^6^ in 1 mL of RPMI 1640 medium, 10% FCS, and rmIL-5 (1 ng/mL; optimal concentration, as previously defined [29]) in 48-well clusters, and incubated at 37°C, 5% CO_2_/95% air, for 7 days. Eosinopoiesis in liquid culture was strictly dependent on IL-5, and culture conditions were adequate for demonstrating both enhancing and suppressive effects [9, 29, 30]. Cells present in 7-day culture were resuspended, collected, counted, cytocentrifuged, and stained for eosinophil peroxidase (EPO; cyanide-resistant peroxidase), a murine eosinophil lineage-specific marker, present from the earliest precursors to terminally differentiated eosinophils [27, 28], and scored as detailed in [9].

### 2.6. Statistical Analyses

Data (mean ± SEM) were analyzed by factorial analysis of variance with the Tukey HSD correction for groups of equal size, using Systat for Windows version 4 software from Systat Inc. (Evanston, IL) [10]. For groups of unequal size, the Bonferroni correction was used [9]. *P* < 0.05 was considered significant.

## 3. Results

### 3.1. 5-LO Deficiency Abolishes the Hemopoietic Response to Allergen Challenge in Sensitized Mice

**Figure 1:**
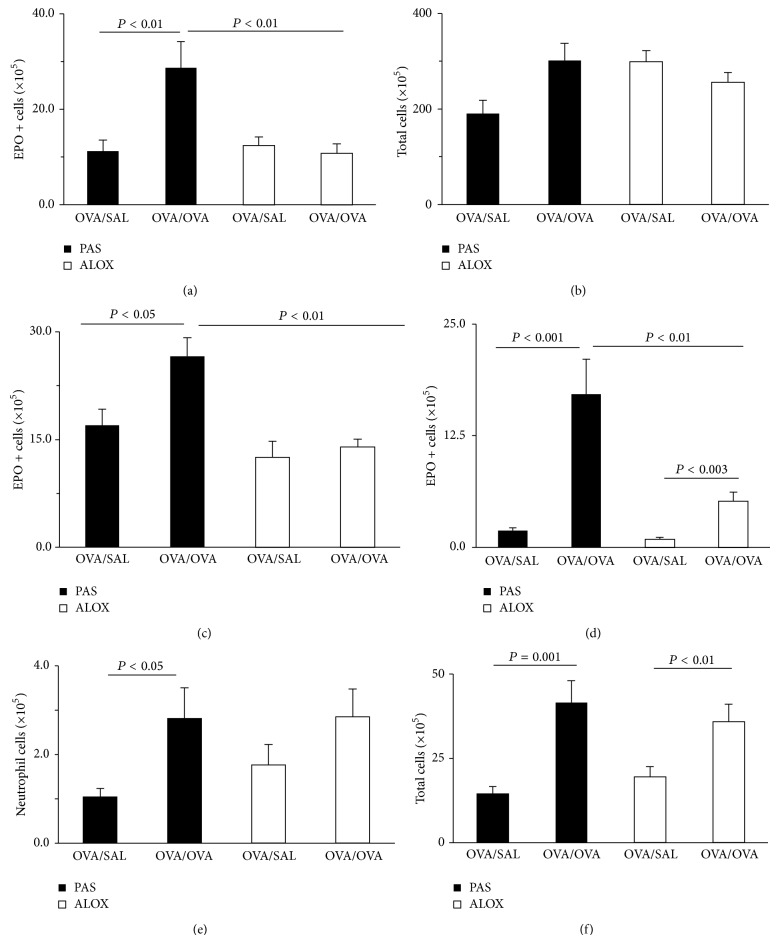
Effect of 5-LO deficiency on the eosinophilia of bone-marrow* in vivo* in OVA-sensitized and -challenged mice. PAS and ALOX mice were sensitized with OVA and challenged i.n. (a, b) or i.p. (c–f) with saline (SAL) or OVA. Bone-marrow was harvested 48 h after challenge. Data for (a) and (b) (mean + SEM; *n* = 6/7/9/9 in the four groups, from left to right) show (a) numbers of EPO+ cells in freshly collected femoral bone-marrow; (b) numbers of total nucleated cells in the same samples. Data for (c) and (d) (mean + SEM; *n* = 7/8/8/8) show (c) numbers of EPO+ cells in bone-marrow; (d)–(f) numbers of EPO+ cells (d), neutrophils (e), and total leukocytes (f) in peritoneal lavage fluid. All significant differences are indicated.

We initially examined whether the integrity of the 5-LO pathway was required for allergen challenge to induce an increase in bone-marrow eosinophil production.  Figure 1 shows the results of sensitization and intranasal ((a)-(b)) or intraperitoneal ((c)–(f)) challenge with OVA in 5-LO-deficient ALOX mice and the wild-type controls (PAS) of the same genetic background. In PAS mice, the number of EPO+ cells in freshly harvested femoral bone-marrow from OVA/OVA was significantly increased (Figure 1(a)), relative to the OVA/SAL controls, as had previously been reported for other inbred mouse strains (BALB/c and C57BL/6). By contrast, in ALOX mutants sensitized and challenged with OVA, there was no significant increase in day 0 EPO+ cell numbers, relative to the respective OVA/SAL controls (Figure 1(a)). The response in PAS mice was eosinophil-lineage selective, because we did not observe significant differences in the numbers of total bone-marrow nucleated cells (all lineages considered) in these conditions (Figure 1(b)).

We next examined whether a requirement for 5-LO was also demonstrable when challenge was done through the intraperitoneal route, with no involvement of the airways. PAS mice presented, in response to i.p. OVA challenge, a significant increase in the number of EPO+ cells in bone-marrow, in comparison with OVA/SAL controls (Figure 1(c)). In addition, i.p. challenge induced, as expected, eosinophil accumulation in the peritoneal cavity of challenged PAS mice, which was significant (*P* < 0.001), relative to the OVA/SAL controls (Figure 1(d)). By contrast, OVA-challenged ALOX mice showed no significant increase in the numbers of eosinophils in freshly harvested bone-marrow, relative to the respective OVA/SAL controls (Figure 1(c)). Nevertheless, ALOX mice presented significant accumulation of eosinophils in the challenge site (peritoneal cavity), in comparison with the same controls (Figure 1(d)). Despite the statistical significance of the response, its magnitude amounted to no more than one-third of that found in the wild-type controls. Importantly, this protocol allowed us to distinguish challenge effects on the bone-marrow from those in the peripheral challenge site, as the latter were decreased, but not abolished, by 5-LO deficiency, in sharp contrast to the former.

Because neutrophils are also included in the infiltrating population and are known to respond to 5-LO-derived chemoattractants, such as LTB4 [26], we also examined the neutrophil counts in peritoneal lavage fluid of mice in these experimental groups. There was a significant increase in neutrophil counts relative to unchallenged controls in i.p. challenged PAS controls, but not in ALOX mice (Figure 1(e)). This shows that 5-LO-deficiency, as expected, affects the migration of neutrophils, in addition to migration of eosinophils [26]. The total cell counts, which include a major component of mononuclear phagocytes (monocytes/macrophages [26]), with very few lymphocytes, were significantly increased by allergen challenge in both PAS and ALOX mice (Figure 1(f)), showing that the decrease in eosinophil and neutrophil migration is not due to a general failure of leukocyte recruitment.

To rule out the possibility that these observations were somehow dependent on the microscopic readout system, which involves a human observer, we performed additional controls using an automated assay for eosinophil peroxidase [9]. As previously reported, measurement of EPO activity is in excellent agreement with microscopic scoring of EPO+ cells (not shown).

**Figure 2:**
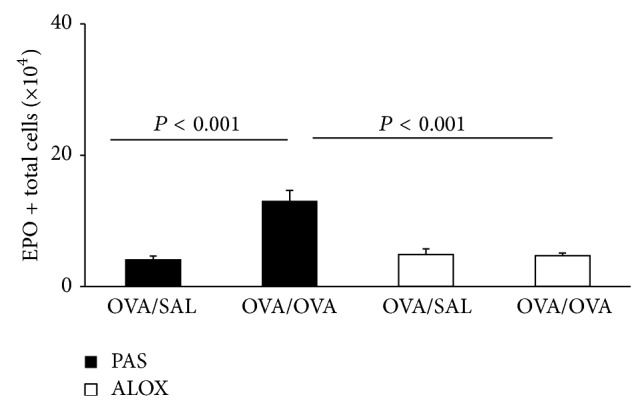
Effect of 5-LO deficiency on the* in vivo* priming of bone-marrow precursors for increased response to IL-5* ex vivo*. PAS and ALOX mice were sensitized with OVA and challenged i.p. Bone-marrow was collected 48 h after challenge and cultured. Data (mean + SEM) are the numbers of EPO+ cells produced in culture with IL-5, 1 ng/mL, for 7 days, from bone-marrow of the indicated experimental groups (*n* = 8). All significant differences are indicated.

Priming* in vivo* for an increased* ex vivo* response to the eosinophil-selective growth and differentiation factor, interleukin (IL)-5, is an important component of the hematological response to allergen challenge [9], which parallels* in vivo* eosinophilia but is independently regulated [29]. We examined whether priming was also dependent on the functional integrity of 5-LO. As shown in Figure 2, in PAS mice sensitized and challenged with OVA, eosinophil production in bone-marrow cultures established with IL-5 was significantly increased, relative to the respective OVA/SAL control. By contrast, in ALOX mice, a comparable increase was not observed. The difference in eosinophil production between OVA/OVA mice of both strains was highly significant.

### 3.2. Pharmacological Blockade of the 5-LO Pathway Prevents the Bone-Marrow Response to Challenge

**Figure 3:**
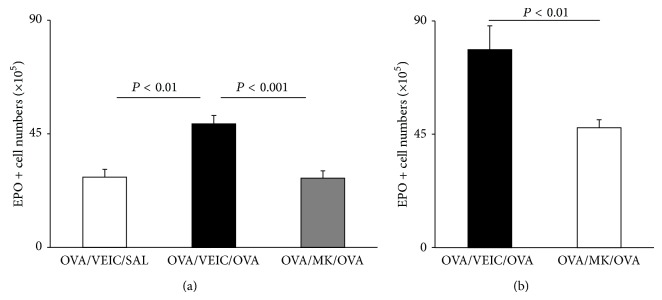
Effect of MK886 on eosinophilia of bone-marrow. BALB/c mice sensitized to OVA were pretreated with vehicle (VEIC, methylcellulose 0,1%) or MK886, 1 mg/Kg, by intragastric administration (500 *μ*L), 24 h and 1 h before challenge by aerosol (a), or by i.p. injection (b). Bone-marrow was collected 24 h (a) or 48 h (b) after challenge. Data (mean + SEM; (a), *n* = 5/5/13; (b), *n* = 3/5) are the number of EPO+ cells in freshly collected femoral bone-marrow after aerosol (a) or i.p. (b) challenge. All significant differences are indicated.

We have examined the relationship of 5-LO to the hematological response to allergen challenge through an independent, pharmacological approach, by using the inhibitor of 5-lipoxygenase activating protein inhibitor, MK886, to block the 5-LO pathway before challenge (Figure 3). BALB/c mice were sensitized with OVA and challenged either by aerosol (Figure 3(a)) or by i.p. injection (Figure 3(b)). Sensitized mice, treated with vehicle before challenge with OVA (OVA/VEIC/OVA), presented a significant increase in EPO+ cell numbers in freshly harvested bone-marrow, relative to the saline-challenged controls (OVA/VEIC/SAL). Treatment with MK886 before OVA challenge (OVA/MK/OVA) prevented the increase in bone-marrow eosinophils in response to allergen challenge, both by aerosol (Figure 3(a)) and by the i.p. route (Figure 3(b)), as shown by the significant differences relative to the respective OVA/VEIC/OVA controls.

### 3.3. The Effectiveness of DEC in Suppressing Eosinopoiesis in Bone-Marrow of Sensitized/Challenged Mice Depends on 5-LO

If the effect of DEC in allergic pulmonary inflammation is mediated by inhibition of leukotriene synthesis,* DEC should be effective in mice which can produce leukotrienes but not in 5-LO deficient animals*, showing that its effectiveness does not involve a secondary pharmacological mechanism unrelated to 5-LO. We initially tested this hypothesis using repeated challenge over a 3-day period, at the end of a 12-day course of DEC, because these are the conditions in which DEC activity was originally demonstrated [20].

**Figure 4:**
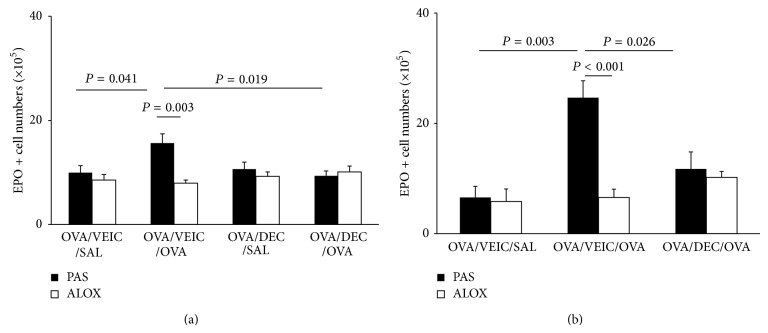
Response to DEC* in vivo* in PAS and ALOX mice sensitized and challenged with OVA. PAS and ALOX mice were sensitized to OVA by two injections 14 days apart in (a) and 7 days apart in (b). DEC (12 mg/kg, 200 *μ*L volume) was given orally in water (vehicle, VEIC), 2 h before challenge. Mice were challenged (a) by the i.n. route (3x, 25 *μ*g/25 *μ*L) and in (b) by aerosol (1x, 2,5% grade II OVA/PBS 1x, for 1 h). Bone-marrow was harvested 24 h after the last challenge. Data (mean + SEM) show the numbers of EPO+ cells in freshly harvested bone-marrow from (a) i.n. challenged mice (PAS *n* = 8/9/5/8; ALOX *n* = 8/7/8/8); aerosol-challenged mice (PAS *n* = 4/5/4; ALOX *n* = 3/5/5). All significant differences are shown.

Figure 4 shows the effect of DEC treatment on the bone-marrow response to allergen challenge, in wild-type PAS controls and in mutant ALOX mice. In PAS mice submitted to the repeated i.n. challenge (Figure 4(a)), there was a significant increase in the numbers of EPO+ cells in freshly harvested bone-marrow from OVA/VEIC/OVA (positive) donors, relative to the OVA/VEIC/SAL unchallenged (negative) controls. Importantly, this increase was abolished by DEC pretreatment (*P* = 0.019 for the difference between OVA/DEC/OVA and OVA/VEIC/OVA). By contrast, ALOX mice did not show an increase in bone-marrow eosinophilia even after repeated challenge, nor a significant change from the baseline when pretreated with DEC. While this shows that PAS mice behave like other strains (BALB/c, C57BL/6) previously shown to respond to DEC in these experimental conditions [20], ALOX mice, which are from the same background but have no functional 5-LO, show absolutely no detectable hematological response to challenge nor to DEC.

These observations were extended to the aerosol challenge model (Figure 4(b)), with essentially identical results. PAS controls showed significant responses to challenge (*P* < 0.003 for the difference between positive and negative controls and *P* < 0.026 for the difference between DEC treatment and the positive control). DEC treatment in saline-challenged controls had no effect of itself, as shown before [20]. Again, ALOX mice showed no increase in eosinophil numbers following challenge, nor a significant response to DEC in any direction.

Together, these observations establish that DEC, in these experimental conditions, is* effective in the presence of 5-LO and has no detectable effect in the absence of 5-LO*, which is consistent with the hypothesis of a mechanism involving leukotriene synthesis inhibition, as opposed to the hypothesis of a secondary (i.e., 5-LO-independent) pharmacological target.

We further examined whether DEC would have an impact on eosinophil numbers in the spleen, which contains large numbers and leukocytes, and is, in specific circumstances, capable of supporting extramedullary hemopoiesis. We did that using the strain (BALB/c) and protocol (i.n. challenge over a 3-day period) originally used to demonstrate the effect of DEC on allergen-stimulated bone-marrow eosinophilia [24], to be sure that DEC was effective on bone-marrow in the experimental conditions used to examine the spleen. Unexpectedly, the numbers of EPO+ cells in spleen of these animals were significantly affected by both allergen challenge and DEC, although in opposite ways.

**Figure 5:**
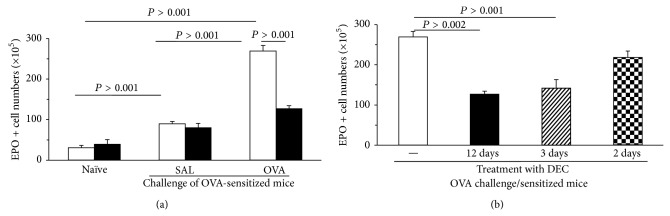
Large-scale eosinophil accumulation in the spleen of sensitized/challenged BALB/c mice is prevented by DEC. Mice were sensitized by two s.c. injections of 100 *μ*g OVA/1.6 mg alum in 0.4 mL saline, at days 0 and 14. Challenge was done i.n. once daily, from days 19 to 21, with OVA (25 *μ*g in 25 *μ*L saline). DEC was given (12 mg/kg/d intragastrically, in 0.2 mL water, (a) and (b)) over a 12-day period, which overlapped in its 3 final days with the challenge schedule, DEC being given 2 h before challenge. (b) DEC was also given as a 3-day course, fully overlapping with the challenge schedule, 2 h before challenge, or as a 2-day course, beginning 1 day after a single unprotected challenge. White bars in (a) and (b), vehicle (water) controls. Black, hatched, and stippled bars in (a) and (b), animals treated with DEC as indicated. Spleens and bone-marrow (not shown in the figure) were collected 24 h after challenge and EPO+ cell counts performed on single cell suspensions of individual mice. Data are mean + SEM of EPO+ cell numbers in the spleens (*n* = 3, all groups). All significant differences are shown.

Intranasal challenge induced a large increase in the eosinophil counts from the spleen of sensitized BALB/c mice (Figure 5).* These represented more than 5-fold the eosinophil counts in the femoral bone-marrow of the same animals. *This shows that, in sensitized and challenged mice of this strain, the spleen accumulates, over a 3-day period a large population of eosinophils, which to our knowledge has not been previously described. Most interesting, this expansion of the splenic eosinophil pool as a function of allergen exposure in the airways was preventable by DEC pretreatment, as previously reported for the main hemopoietic site, bone-marrow [23]. Furthermore, the effect of DEC on spleen eosinophil counts showed the same pattern as the effect of DEC on bone-marrow eosinophilia (Figure 5(b)), because (a) a shorter course of DEC, given only during the challenge period, and preceding each challenge, was as effective as the traditional 12-day course; (b) the effectiveness of DEC would be lost if a single challenge exposure had taken place without DEC pretreatment.

### 3.4. Effect of the CysLT Type I Receptor Antagonist, Montelukast, on Bone-Marrow Response to Challenge

**Figure 6:**
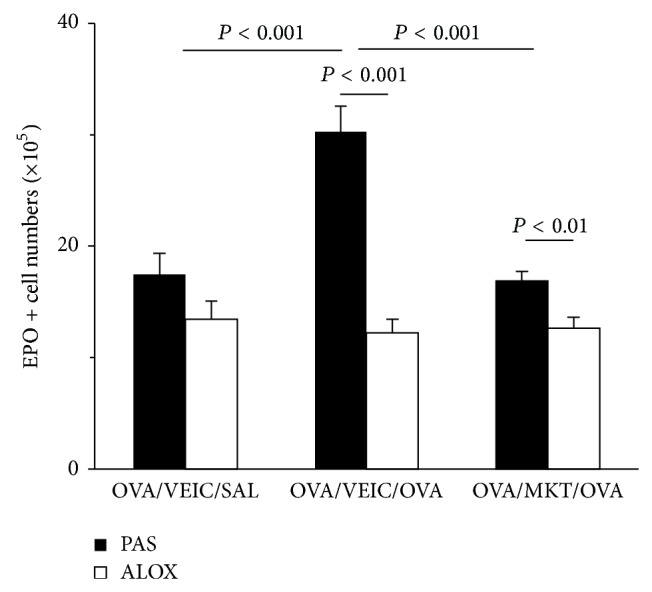
Montelukast blocks the effects of allergen challenge on wild-type bone-marrow but has no effect on 5-LO-deficient bone-marrow. PAS and ALOX mice sensitized to OVA were pretreated with vehicle (VEIC, 2% DMSO/PBS 1x) or montelukast (MKT, 10 mg/Kg), by intragastric administration (500 *μ*L), 1 h before challenge by aerosol. Bone-marrow was collected 24 h after challenge. Data (mean + SEM; PAS, *n* = 8/11/12; ALOX, *n* = 9/11/12) are the number of EPO+ cells in freshly collected femoral bone-marrow after aerosol challenge. All significant differences are indicated.

In view of the effectiveness of 5-LO inactivation/blockade, on the one hand, and of DEC, on the other hand, in preventing eosinophilia in this model, both inside and outside the bone-marrow, it is important to establish whether targeting CysLT effects with a CysLT type I receptor antagonist would be just as effective. This was done in PAS mice and in ALOX mutants (Figure 6). Montelukast abolished the increase in bone-marrow eosinophil counts induced by allergen challenge of PAS mice. By contrast, it had no effect on eosinophil counts in sensitized and challenged ALOX mutants. This shows that montelukast is as effective as deletion of 5-LO in preventing the hematological response to allergen challenge and that its effectiveness, as expected, depends on the presence of CysLT, which cannot be made in ALOX mice.

## 4. Discussion

In this study, we reexamined the relationship between 5-LO function, the hematological response to sensitization and challenge in allergy models, and the effectiveness of DEC in these conditions, by a series of complementary approaches. We tested the hypothesis that if DEC was acting through leukotriene synthesis inhibition, its effects [20] would be duplicated in murine bone-marrow by inactivation of the leukotriene biosynthetic pathway, as well as by other drugs acting on the same target. Overall, there is an excellent agreement between the observations made through these distinct approaches, and an important role for the 5-LO pathway in the hematological response to allergen challenge in mice was established.

The same experiments showed that this is a general phenomenon, not restricted to airway challenge and allergic pulmonary inflammation, and further provided evidence that the eosinophilia of the bone-marrow (central) and of the peritoneal cavity (peripheral) can be dissociated in specific experimental conditions.

### 4.1. Agreement and Complementarity of Gene Inactivation and Pharmacological Blockade Approaches

ALOX mutants and the PAS mice of the same background provided an excellent combination to assess the effect of 5-LO inactivation [26] and were very useful in confirming the relationship of drug effects to the presence of a functional 5-LO.

For inhibition of the 5-LO pathway, we chose MK886 because it is considered specific [14, 15] and does not have an effect on the bone-marrow by itself [19, 20]. Importantly, neither MK886 treatment nor 5-LO inactivation changed significantly the baseline of EPO+ cell numbers in bone-marrow, showing that neither condition affects steady-state eosinophil production. This is consistent with previously published studies on eosinophils from ALOX mice* in vivo* [26] as well as* in vitro* [19, 20]. Hence, the effect of 5-LO inactivation/blockade was significant only for the selective increase in eosinopoiesis inside bone-marrow from sensitized mice induced by* in vivo* allergen exposure, which is the primary effect of DEC in murine allergy [24].

By contrast, that IL-5 is necessary for both the steady-state (baseline) production and the increased production prompted by an immune response, as documented by the classical study of Nishinakamura and colleagues [21]. Hence, 5-LO and IL-5 play complementary and distinct roles, with IL-5 being the indispensable lineage-selective growth factor, while 5-LO is necessary for the modification of IL-5 effects, which ultimately result in an increased eosinophil production.

The usefulness of combining genetic and pharmacological approaches is demonstrated in this study by our ability to establish a link between 5-LO and the effectiveness of DEC and montelukast in blocking the hematological response to challenge. This was accomplished by showing an effect for both drugs in wild-type mice, which respond to allergen challenge,* together with* the absence of any effect in mutants lacking 5-LO, which do not respond to challenge. The conclusion was based on a* positive *result (blockade of the response to allergen challenge in wild-type mice), together with a* negative* control result (no effect of any of the drugs in mice lacking their putative pharmacological target, namely, the 5-LO pathway).

The inclusion of a negative control branch in these experiments may seem unnecessary or even exaggerated, as the drugs we used could reasonably be assumed to have no effect in an animal which lacks the physiological response to challenge that they are expected to block. However, caution is recommended when a drug, or a drug panel, is evaluated, because many drugs have been shown in the past to have unexpected actions, due to effects on previously uncharacterized pharmacological targets, distinct from those assumed by the investigators to be relevant. Our results are reassuring, indeed. Neither diethylcarbamazine nor montelukast had any significant impact on eosinophil production in mice lacking 5-LO. For montelukast, the results support the assumption that it is selective for CysLT1 receptors. For diethylcarbamazine, which has biochemical effects other than inhibition of leukotriene production [31], we feel more confident about its dependence on 5-LO because it had no effect in ALOX bone-marrow.

### 4.2. Involvement of CysLT* In Vivo*

Furthermore, we have examined whether montelukast would duplicate the effects of MK886 and of 5-LO inactivation, as expected if CysLT were the relevant 5-LO products lacking in the presence of MK886 or an inactive 5-LO. The results show that montelukast is as effective in wild-type mice as MK886 and, like DEC, does not work in ALOX mutants. This is consistent with the effect of allergen challenge in mice being accounted for by CysLT, as suggested by previous human studies, and argues against an important role for LTB4 or lipoxins, which are not counteracted by CysLT receptor blockade, in this response. A further prediction is that mice lacking CysLT type I receptors should behave as mice pretreated with montelukast and show no eosinopoietic response to allergen challenge, an issue that should be addressed in future studies.

### 4.3. Priming* In Vivo*

Priming of bone-marrow* in vivo* for an increased* ex vivo* response to IL-5 in bone-marrow culture is a very reproducible effect of allergen challenge [9, 10]. Allergen challenge of sensitized animals in different models has been shown to result in IL-5 [9, 10, 12] and CysLT production [13, 15]. Montelukast, which blocks CysLT actions on eosinopoiesis* in vitro* [19, 20], acted* in vivo* to block the primary effect of allergen challenge on the bone-marrow, which is an increased eosinophil count, usually accompanied by priming for increased responses to IL-5 in culture [9, 10]. Hence, it is reasonable to assume that priming involves an effect of CysLT that can be blocked by montelukast and is therefore mediated by CysLT1R.

Priming in our experiments took place in the 48 h period after challenge, during which bone-marrow is likely to have been exposed to both IL-5 and CysLT. So, CysLT do not prime eosinophils in the absence of IL-5 for an increased response to a subsequent exposure to IL-5. Instead, the exposure of bone-marrow* in vivo* to both IL-5 and CysLT primed for an increased exposure to IL-5 alone. Importantly, both IL-5 [9] and 5-LO/CysLT (as shown here) are required for the hematological response to allergen challenge. This means that selective increases in eosinopoiesis can be accounted for by a synergic combination of IL-5 and CysLT, in which IL-5 is eosinophil-selective, while CysLT act on cells stimulated by IL-5 to enhance IL-5 effects.* Because the target of CysLT depends on IL-5 to be stimulated, the synergic combination of CysLT and IL-5 necessarily works as lineage-selective stimulus.*

One should be cautious when comparing the above situation with that in bone-marrow cultures established from naive (nonsensitized) mice [19, 20]. It has been shown that CysLT, when added* in vitro* together with IL-5, significantly enhance the production of eosinophils in bone-marrow culture [19, 20]. In these conditions, CysLT are ineffective in the absence of IL-5, as they do not support eosinophil production in bone-marrow culture by themselves (unpublished observations). While priming can be studied* in vitro*, by separate addition to the cultures of the priming agent (CysLT) and the growth factor (IL-5), this is probably informative only for the* in vitro* situation, where one can control the time of exposure to (exogenous) leukotriene and IL-5. This experimental design cannot be extrapolated to the analysis of events* in vivo*, where such manipulation cannot be done without changing the underlying conditions of the entire study.

### 4.4. Extramedullary Effects

Finally, an unexpected finding in the study of the relationship of DEC to 5-LO was its effectiveness against the large-scale accumulation of eosinophils in the spleen of sensitized/challenged BALB/c mice, which is induced by challenge, and therefore builds up in the course of 3 days (the repeated challenge period). Several lines of evidence indicate that the accumulation of eosinophils in these two sites (spleen and bone-marrow) is part of an integrated hematological response to sensitization and challenge. Eosinophilia in both sites is (a) induced by challenge in BALB/c mice over the same three-day period; (b) prevented with identical effectiveness by long-term (12-day) and short-term (3-day) courses of DEC; and (c) resistant to DEC treatment after a single unprotected challenge [20].

The magnitude of the eosinophil response in the spleen is itself surprising, as it largely surpasses the counts in femoral bone-marrow, suggesting that after challenge* the spleen may quickly become the largest reservoir of eosinophils in vivo*, through a leukotriene-dependent mechanism, which clearly deserves further examination, by its magnitude as well as by its fast responsiveness to DEC therapy.

This phenomenon, to our knowledge, has not been described in a murine model of allergic disease. However, there have been reports of splenic eosinophilia in humans, associated with fatal anaphylactic reactions, which are paralleled by massive mast cell degranulation in splenic tissue, a finding that suggests that the spleen is a major shock organ in systemic anaphylaxis [32]. If so, the findings in the murine model suggest that massive accumulation of eosinophils in the spleen also occurs during nonfatal allergic reactions and is dependent on 5-LO and responsive to DEC treatment. This should prompt future examination of the possible benefit of DEC treatment in models of systemic anaphylaxis.

### 4.5. Relationship to iNOS

One important implication of these findings is that the mechanism of DEC actions in the hematological response to allergen challenge must be reevaluated, taking into account that it requires 5-LO. Previous studies [24, 25] had shown DEC effects to be dependent on inducible NO synthase, which is an essential part of a proapoptotic pathway activated by a variety of soluble ligands [30]. The present study makes it unlikely that DEC induces or activates iNOS directly, as no effect of DEC was observed in 5-LO-deficient mice. However, there may be an indirect relationship of DEC to iNOS such that iNOS activation or expression in the bone-marrow of sensitized/challenged mice requires blockade of 5-LO. This should be examined in the future, by direct monitoring of iNOS and 5-LO in bone-marrow exposed to the same panel of drugs used in this study.
